# Are Genetic Therapies for Epilepsy Ready for the Clinic?

**DOI:** 10.1177/15357597231176234

**Published:** 2023-05-19

**Authors:** James S. Street, Yichen Qiu, Gabriele Lignani

**Affiliations:** 1 Department of Clinical and Experimental Epilepsy, UCL Queen Square Institute of Neurology, London, United Kingdom

**Keywords:** genetic therapies, epilepsy, genetic epilepsies, refractory epilepsy

## Abstract

In recent years, there has been a significant increase in preclinical studies to test genetic therapies for epilepsy. Some of these therapies have advanced to clinical trials and are being tested in patients with monogenetic or focal refractory epilepsy. This article provides an overview of the current state of preclinical studies that show potential for clinical translation. Specifically, we focus on genetic therapies that have demonstrated a clear effect on seizures in animal models and have the potential to be translated to clinical settings. Both therapies targeting the cause of the disease and those that treat symptoms are discussed. We believe that the next few years will be crucial in determining the potential of genetic therapies for treating patients with epilepsy.

Epilepsy is one of the most common severe neurological diseases with many patients having refractory seizures without any effective options for treatment. Recently, several genetic therapies have been developed with the aim of treating this intractable population of patients. Among these approaches some are already in or nearing clinical trials, bringing hope to the epilepsy field and to the patient community. These treatments can be divided in two broad categories: genetic therapies targeting the cause of the epileptic phenotype, and those aiming to address the symptoms, namely the seizures (Table 1).

**Table 1.** Current Genetic Therapies for Epilepsy Preclinically Tested.

	Strategy	Disease targeted	Vector delivered	Clinical trial	Citations
Causes	Gene supplementation	Dravet syndrome	HC-AdV-CAG-SCN1A/pAAV9-pGad1-NaVβ1-myc	–	1,2
		*CDKL5* deficiency disorder	AAV-PHP.B-CBh-hCDKL5_1	–	3
	Gene modulation	Dravet syndrome	AAV9-PhP.eB-TetOn/mdlx-dCas9A-Scn1a/AAV9-PhP.eB-flox-VGAT-dCAS9A-Scn1a/AAV9-RE(GABA)-eTF(Scn1a)	ENDEAVOR (NCT05419492)	4-6
	Antisense oligonucleotides	Dravet syndrome	ASO-Scn1a nonproductive splicing event	MONARCH (NCT04740476)	7,8
		*SCN2A* DEE	ASO-Scn2a	–	9
		*SCN8A* encephalopathy	ASO-Scn8a	–	10
		Epilepsy of infancy with migrating focal seizures	ASO-Kcnt1	–	11
		Lafora disease	ASO-*Gys1*	–	12
		Angelman syndrome	ASO-Ube3a-ATS	GTX-102 (NCT04259281)	13,14
	RNA interference	Lennozx-Gastaut syndrome	scAAV9-miDnm1a	–	15
Symptoms	Neuropeptides	TLE	scAAV2-CBA-pDyn (predynorphin)	–	16,17
		TLE	rAAV1/2-CBA-NPY/AAV1-CAG-NPY-hY2	–	18,19
		TLE	AAV2-FIB-GAL (Galanin)	–	20
	Exogenous proteins	TLE and neocortical focal epilepsy	AAV5-CamkIIα-HA-hM4D(Gi)/AAV2/5-hCAMKII-hM4D(Gi)/AAV2/7-CamKIIα-hM4D(Gi)/AAV2.1-hSyn-hM4Di	–	21-23
		TLE, neocortical focal epilepsy and cortical injury	LV-Camk2a-NpHR/rAAV5-Camk2a-eNpHR/CamKII/PV-cre x floxed-STOP ChR	–	24-28
		Neocortical focalepilepsy	Lentivirus CAMK2A-eGluCL	–	29
	Endogenous channels	TLE	AAV9 CamKII-dCAS9A-Kcna1	–	30
		TLE and neocortical focal epilepsy	Lenti/AAV9 CAMK2A-EKC(KCNA1)	NCT04601974	31,32
		TLE	AAV9-cfos-EKC (KCNA1)	–	33
		Episodic ataxia type 1 with epilepsy	ASO-Scn8a	–	34
		Dravet syndrome	ASO-Scn8a	–	10
		*KCNQ2* DEE	ASO-Scn8a	–	34
		TLE	AAV10-shRNA-Scn8a	–	35

## Treating the Cause

Although epilepsy overall shows strong heritability, there are several, individually rare, monogenic syndromes. These are commonly *de novo*, although can also be inherited in a dominant or recessive manner. Among causative genes are several ion channels (*SCN1/2/8A*, *KNCA1*, *KCNQ2*/*3*, *GABRA1, KCNT1*), mutations of which are associated with the umbrella class of developmental and epileptic encephalopathies (DEEs). The genetic background of DEEs and their resistance to conventional anti-seizure therapies identify these diseases as obvious candidates for genetic therapy. By modifying the expression or activity of the implicated gene products, the goal of such therapies is to correct the underlying molecular pathology.^36^

### Gene Supplementation

Loss-of-function mutations (LoF) are in principle treatable with gene supplementation of a healthy copy of the mutated gene, for instance using a viral vector. Indeed, gene supplementation has been successfully trialed in mouse models of Cyclin-dependent kinase-like 5 (*CDLK5*)-deficiency disorder, an X-linked DEE caused by *de novo* LoF mutations in the *CDKL5* gene. Gene supplementation with a human *CDKL5*, delivered with an adeno-associated viral vector (AAV), successfully ameliorated the disease phenotype of *Cdlk5* knock-out mice.^3^ Recent work has also demonstrated the viability of gene supplementation to improve outcomes in mouse models of Dravet syndrome, a genetic DEE frequently caused by haploinsufficiency of the voltage-gated sodium ion channel Nav1.1 secondary to *de novo* mutations in *SCN1A.* Adenovirus (AdV)-mediated delivery of *SCN1A* reduces spontaneous seizures and mortality in Dravet syndrome mouse models, as does AAV-mediated delivery of *SCN1B* (the beta-subunit of Nav1.1, which is smaller and has been shown to promote trafficking of alpha subunits to the cell surface).^1,2^ Although gene supplementation is a straightforward approach, it is limited by several considerations: AAV vectors have a restricted packaging capacity, which is exceeded by the full-length *SCN1A* open reading frame; conversely, AdV vectors do not spread widely in the central nervous system (CNS) and are potentially toxic; finally, it is unclear which neurons and which brain regions need to be treated, and at what developmental stage, in order to achieve an optimal therapeutic effect.

### Gene Modulation

Ion channels are among the most important genes implicated in neurodevelopmental epilepsies but are often too large for straightforward gene supplementation. To overcome this obstacle, a modified version of the gene editing tool CRISPR/Cas9, known as CRISPR activation (CRISPRa), has shown potential in treating Dravet syndrome. Using CRISPRa targeting of the *SCN1A* promoter, it is possible to upregulate the expression of *SCN1A*. Accordingly, a recent paper has demonstrated that AAV delivery of CRISPRa constructs into a mouse model of Dravet syndrome increases Nav1.1 protein levels, rescues excitability of cortical parvalbumin-positive (PV+) neurons, and reduces susceptibility to thermally induced seizures otherwise observed in the model.^4^ Another study achieved a therapeutic effect in a mouse model of Dravet syndrome crossed into a mouse line constitutively expressing most of the CRISPRa machinery.^5^ In both of these studies, the transcriptional upregulation was biased to inhibitory interneurons, which fail to tolerate SCN1A haploinsufficiency, and the anti-epileptic effect is thought to be mediated by an increase in their excitability.

Interestingly, a similar approach using defective Zinc Finger Nucleases fused to a transcriptional activator targeted to the *Scn1a* promoter has also been shown to be effective in a mouse model of Dravet syndrome. Another study achieved upregulation of *SCN1A* in inhibitory interneurons using an AAV to express a GABArgic cell-selective transcription factor, resulting to an increase in Nav1.1 protein expression.^6^ Additional safety studies in nonhuman primates (NHPs) showed good uptake of the construct throughout the brain and with no adverse events.^6^ In principle, these approaches can be used to upregulate any gene, and thus treat a range of LoF mutations causing epilepsy, although implementation requires substantial bioinformatic insights into promoter and enhance function.

### Antisense Oligonucleotides and RNA Interference

Several *de novo* mutations causing epilepsy in ion channels are gain-of-function (GoF) and so are not amenable to gene supplementation. For these mutations decreasing expression at the gene or messenger RNA (mRNA) level could potentially have a therapeutic role. Antisense oligonucleotides (ASOs) are short single-stranded nucleic acid sequences, typically modified to increase their stability, which are capable of targeting an mRNA transcript of interest to modulate pre-mRNA splicing events, or regulate translation, thus modulating the efficiency of transcript processing and the production of a protein of interest. In recent years, ASOs have been shown to ameliorate epileptic phenotypes in murine models of several GoF DEEs. *Scn2a* ASOs have been administered to *Scn2a* mutant mice, reducing seizure frequency, and extending life span.^9^ Similarly, reduction of *Scn8a* transcripts using ASOs has been shown to improve the frequency of behavioral and electroencephalographic seizures and overall survival of *Scn8a* mutant mice.^10^ Antisense oligonucleotides therapy has further been shown to improve outcomes in mouse models of other epilepsy-associated syndromes, including *KCNT1*-associated epilepsy of infancy with migrating focal seizures,^11^ myoclonic epilepsies such as Lafora disease,^12^ and Angelman Syndrome.^13,14^ It has also been shown to reduce aberrant transcript expression in patient-derived fibroblasts in Unverricht-Lundborg disease.^37^

Importantly, while ASOs typically knock-down transcript activity, they also have potential for upregulating gene expression, making them suitable for use in LoF genetic variants. Targeted augmentation of nuclear gene output (TANGO) aims to increase target gene expression by inhibiting nonproductive mRNA splicing events,^38^ with recent work demonstrating a successful increase in productive *Scn1a* transcripts. This was associated with a concurrent reduction in seizure frequency and fatality following ASO-TANGO treatment in a mouse model of Dravet syndrome.^7^ A similar method used an oligonucleotide-derived compound (AntagoNAT) to knock-down antisense noncoding RNA that suppresses *Scn1a* expression. This strategy led to an increase in *Scn1a* transcript in mice and African green monkeys, and an amelioration of the *Scn1a*-mediated epileptic phenotype in Dravet mice.^8^

Another way to downregulate mRNA levels is using short-harpin RNAs (shRNA) or micro RNAs (miRNA). These tools have shown promise for improving the phenotype of some genetic epilepsies, including dynamin-1 associated epileptic encephalopathy.^15^

These therapeutic approaches are very promising, and they have the advantage of targeting mRNA, therefore avoiding expression modulation in cells not expressing the gene of interest. However, one potential limitation is that the transcriptional modulation is not permanent, potentially requiring retreatment in a clinical setting.

## Treating the Symptoms

For the overwhelming majority of patients, there is no single identifiable underlying molecular defect at the root of seizures. In cases of refractory focal epilepsy with a defined focus, treating the symptoms (the seizures) is a feasible alternative to resective surgery. Seizures lead to major disruptions in patients’ lives and significantly increase the risk of sudden unexpected death in epilepsy. Gene therapies have a potential role in manipulating the epileptic network and to suppress seizures, usually by either promoting inhibition or suppressing excitation. Many different approaches have been developed so far with some very close to first in-human clinical trials.

### Neuropeptides

Neuropeptides are attractive therapeutic targets for their highly specific interactions with target receptors and very low effective concentrations.^39^ One of the promising neuropeptides is dynorphin, which interacts with kappa opioid receptors and exerts an inhibitory effect in the limbic system.^40^ Adeno-associated viral vector delivered pre-pro-dynorphin in hippocampal regions in rat and mouse chronic models of epilepsy leads to a prolonged suppression of spontaneous seizures, and prevents a decline of learning and memory.^16,17^

Neuropeptide Y (NPY) is a secreted neuropeptide widely expressed in the central nervous system, especially in the limbic system, including hippocampus and amygdala. Overexpression of a human NPY in the hippocampus using an AAV has shown an anti-seizure effect (40% reduction in seizure frequency).^18^ This effect was most likely mediated by the inhibition of presynaptic glutamate release via NPY receptor Y2 (NPY-Y2).^41^ Indeed, the combined overexpression of NPY and NPY-Y2 with a single vector (CG01) showed a more prominent anti-seizure effect.^19^

Another neuropeptide implicated in modulating glutamate release is galanin.^42^ AAV-mediated overexpression of galanin has shown to decrease evoked seizure threshold.^20^

### Engineered Channels

The modification of receptors and channels has been used not only to understand basic neurophysiology but also to treat neurological diseases modulating neuronal properties. Both optogenetic activation of inhibitory neurons and optogenetic inhibition of excitatory neurons have been shown to decrease epileptic activity in different rodent models, with several brain regions targeted including cortex, hippocampus, cerebellum, midbrain, and thalamus.^24-28,31^

Clinical translation of optogenetics presents substantial challenges, not least because of the requirement to deliver light to the brain. Although acting with a lower temporal resolution, chemogenetics can also be used for on-demand or controllable modulation of neural circuits. The activity of neurons within the epileptic focus can be controlled, for instance, by using designer receptors exclusively activated by designer drugs. AAV-delivered inhibitory hM4D(Gi) receptors targeted to excitatory neurons, when activated by clozapine metabolite clozapine N-oxide or olanzapine,^43^ showed robust anti-seizure effects.^21,22^ Recently, a decrease in cortical seizure severity using AAV-hM4D(Gi), coupled with systemic injection of deschloroclozapine, has been shown in NHPs.^23^

Another approach to chemogenetics that dispenses with the need for an exogenous ligand to activate the synthetic receptor is to modify the biophysical properties of a receptor of interest to respond to endogenous signals related to seizures. Recently eGluCl (glutamate-gated chloride channel with enhanced glutamate sensitivity) has been shown to be a promising tool to decrease epileptic events in a rat model of neocortical focal epilepsy, using pathological increases of glutamate in the extracellular space as the endogenous activator of the receptor.^29^

### Endogenous Channels

While exogenous channels could encounter issues with immunogenicity in clinical translation, endogenous channels offer promising alternatives. Reduction of voltage-gated sodium channel Nav1.6 expression, via ASO or shRNA-mediated knockdown of *Scn8a*, compensates for some epileptic features observed in animal models of a variety of ion channel haploinsufficiencies (*Scn1a+/-*, *Kcna1-/-* and *Kcnq2fl/fl* mutant mice) and temporal lobe epilepsy.^10,34,35^ Alternatively, overexpression of the voltage-gated potassium channel Kv1.1 in excitatory neurons in either the neocortex or the hippocampus can suppress spontaneous seizures and epileptiform activity in chronic rodent models of epilepsy.^31,32^ Recently, upregulation of Kv1.1 using CRISPRa has also been demonstrated to reduce seizure frequency and improve behavioral comorbidities in a chronic mouse model of focal epilepsy.^30^ Another approach to overexpressing an endogenous channel is through activity-dependent promoters that follow neuronal dynamics and selectively target hyperexcitable neurons. When delivered to the epileptic focus of a mouse model of chronic epilepsy using AAV, this tool greatly reduces spontaneous seizures. This approach’s advantage lies in its specificity for pathological neurons both in time and space and its self-regulatory mechanisms.^33^

## Roadblocks to the Clinic

For treatments addressing a molecular defect underlying epilepsy to be feasible, the cause must be known and identifiable in patients. However, as mentioned above, monogenic epilepsy is rare, and there is a large degree of heterogeneity in the genetic cause of DEEs, necessitating the use of precision medicine to tailor genetic approaches to individual patients. For instance, while ASO and CRISPRa methods for Dravet syndrome work to increase *SCN1A* expression in the context of LoF-mediated haploinsufficiency, these methods would be contraindicated in a recently reported subset of patients with early infantile epileptic encephalopathy in whom GoF *SCN1A* mutations have been identified.^44^ As precision medicine remains expensive and time-consuming, this introduces significant logistical and financial hurdles before its systematic introduction into the clinic.

Importantly, as these epilepsy syndromes frequently present and progress early in childhood, the question of the most appropriate time of intervention is vital. Genetic interventions may display a temporally narrow window of efficacy, after (or before) which little amelioration of the disease phenotype may be seen.^45,46^ This may be especially the case for therapies targeting ion channels expressed only transiently in development—for instance, *SCN1A*, which peaks at 7 to 9 months of age and decreases in later childhood and adulthood.^47^ Further, once secondary sequelae emerge due to the primary pathology, treatment solely of the cause may be inappropriate to address the full syndrome of deficits observed. While some evidence suggests that reestablishing *SCN1A* expression in older (P30 and P90) Dravet mice reduces spontaneous seizures and rescues some behavioral deficits,^48^ it remains unclear how to extrapolate these findings to patients. It is not possible to predict if such interventions later in life will in general successfully rescue the epileptic phenotype, or its sequelae such as developmental delay, at which point further pathology secondary to the epileptic attacks may be permanently established. These encephalopathic features of DEEs may not be entirely secondary to the epileptic features, with some symptoms in Dravet patients emerging independently of seizures.^49^ In these cases, it remains unclear if genetic corrections designed to induce seizure freedom will also ameliorate the nonepileptic, encephalopathic features of disease.

In spite of these roadblocks, translation to the clinic is imminent. Indeed, clinical trials for genetic therapies to treat Dravet syndrome and Angelman syndrome have begun. The MONARCH study uses ASO-TANGO technology (STK-001, NCT04740476) to upregulate Nav1.1 expression, with preliminary results suggesting that single and multiple doses of STK-001 are well-tolerated and may reduce seizure frequency. In parallel, with the demonstration of a sufficient safety profile of the transcriptional activator ETX101 in NHPs,^6^ the ENDEAVOR trial is a phase I/II clinical trial (NCT05419492) aiming to assess the safety and efficacy of ETX101 in infants and children with Dravet syndrome and is due to begin recruitment this year. Further, a phase I/II clinical trial is ongoing to assess the safety of an intrathecally administered ASO therapy in patients with Angelman syndrome (NCT04259281).

Importantly, treating symptoms remains the only option for many patients without a genetic diagnosis. In practice, it is often difficult to draw clear relationships between genetic landscape and epileptic seizures. Apart from a small proportion of patients with identified familial or *de novo* monogenic mutations, the majority of patients with epilepsy have complex etiologies with potentially multiple genetic contributions.^50^ The high variety of causes of epilepsy, and the likely concurrent high variability of genetic changes in the pathological tissue, complicate the design of any individual genetic targeting tool, especially for epilepsies of nongenetic origin. Even when the genetic cause seems clear, like for Dravet, other genetic variability in patients’ genomes could be pivotal for the development of the pathology.^51^ Further, in cases when a pathology with known genetic cause has been consolidated in patients for many years, targeting the seizures could be a promising option. With ongoing research into epilepsy mechanisms, more potential gene therapy targets are being revealed significantly increasing the possibilities for patients. Remarkably, the first gene therapy for epilepsy symptoms using Kv1.1 is due to start this year and is soon recruiting patients with focal epilepsy for a Phase I/IIa clinical trial (NCT04601974). Other approaches are similarly on the path to be registered for their first in-human clinical trials, ushering in a new era for genetic therapies in epilepsy.

## References

[1] Niibori Y Lee SJ Minassian BA Hampson DR . Sexually divergent mortality and partial phenotypic rescue after gene therapy in a mouse model of Dravet syndrome. Hum Gene Ther. 2020;31(5-6):339–351. 31830809 10.1089/hum.2019.225 PMC7087406

[2] Mora-Jimenez L Valencia M Sanchez-Carpintero R , et al. Transfer of SCN1A to the brain of adolescent mouse model of Dravet syndrome improves epileptic, motor, and behavioral manifestations. Mol Ther Nucleic Acids. 2021;25:585–602. 34589280 10.1016/j.omtn.2021.08.003 PMC8463324

[3] Gao Y Irvine EE Eleftheriadou I , et al. Gene replacement ameliorates deficits in mouse and human models of cyclin-dependent kinase-like 5 disorder. Brain. 2020;143(3):811–832. 32125365 10.1093/brain/awaa028

[4] Colasante G Lignani G Brusco S , et al. dCas9-based Scn1a gene activation restores inhibitory interneuron excitability and attenuates seizures in Dravet syndrome mice. Mol Ther. 2020;28(1):235–253. 31607539 10.1016/j.ymthe.2019.08.018 PMC6952031

[bibr5-15357597231176234] Yamagata T Raveau M Kobayashi K , et al. CRISPR/dCas9-based Scn1a gene activation in inhibitory neurons ameliorates epileptic and behavioral phenotypes of Dravet syndrome model mice. Neurobiol Dis. 2020;141:104954. 32445790 10.1016/j.nbd.2020.104954

[6] Tanenhaus A Stowe T Young A , et al. Cell-selective adeno-associated virus-mediated Scn1a gene regulation therapy rescues mortality and seizure phenotypes in a Dravet syndrome mouse model and is well tolerated in nonhuman primates. Hum Gene Ther. 2022;33(11-12):579–597. 35435735 10.1089/hum.2022.037 PMC9242722

[7] Han Z Chen C Christiansen A , et al. Antisense oligonucleotides increase *Scn1a* expression and reduce seizures and SUDEP incidence in a mouse model of Dravet syndrome. Sci Transl Med. 2020;12(558):eaaz6100. 32848094 10.1126/scitranslmed.aaz6100

[8] Hsiao J Yuan TY Tsai MS , et al. Upregulation of haploinsufficient gene expression in the brain by targeting a long non-coding RNA improves seizure phenotype in a model of Dravet syndrome. EBioMedicine. 2016;9:257–277. 27333023 10.1016/j.ebiom.2016.05.011 PMC4972487

[9] Li M Jancovski N Jafar-Nejad P , et al. Antisense oligonucleotide therapy reduces seizures and extends life span in an SCN2A gain-of-function epilepsy model. J Clin Invest. 2021;131(23):e152079. 34850743 10.1172/JCI152079 PMC8631599

[10] Lenk GM Jafar-Nejad P Hill SF , et al. Scn8a antisense oligonucleotide is protective in mouse models of Scn8a encephalopathy and Dravet syndrome. Ann Neurol. 2020;87(3):339–446. 31943325 10.1002/ana.25676 PMC7064908

[11] Burbano LE Li M Jancovski N , et al. Antisense oligonucleotide therapy for *KCNT1* encephalopathy. JCI Insight. 2022;7(23). 10.1172/jci.insight.146090 PMC9746904 36173683

[12] Ahonen S Nitschke S Grossman TR , et al. Gys1 antisense therapy rescues neuropathological bases of murine Lafora disease. Brain. 2021;144(10):2985–2993. 33993268 10.1093/brain/awab194 PMC8634080

[13] Milazzo C Mientjes EJ Wallaard I , et al. Antisense oligonucleotide treatment rescues UBE3A expression and multiple phenotypes of an Angelman syndrome mouse model. JCI Insight. 2021;6(15):e145991. 34369389 10.1172/jci.insight.145991 PMC8410092

[14] Dindot SV Christian S Murphy WJ , et al. An ASO therapy for Angelman syndrome that targets an evolutionarily conserved region at the start of the UBE3A-AS transcript. Sci Transl Med. 2023;15(688):eabf4077. 36947593 10.1126/scitranslmed.abf4077

[15] Aimiuwu OV Fowler AM Sah M , et al. RNAi-based gene therapy rescues developmental and epileptic encephalopathy in a genetic mouse model. Mol Ther. 2020;28(7):1706–1716. 32353324 10.1016/j.ymthe.2020.04.007 PMC7335739

[16] Agostinho AS Mietzsch M Zangrandi L , et al. Dynorphin-based “release on demand” gene therapy for drug-resistant temporal lobe epilepsy. EMBO Mol Med. 2019;11(10):e9963. 31486590 10.15252/emmm.201809963 PMC6783645

[17] Christian-Hinman CA . Is on-demand dynorphin destined to be in demand to decrease seizures? Epilepsy Curr. 2021;21(1):48–50. 34025273 10.1177/1535759720951791 PMC7863305

[18] Noe F Pool AH Nissinen J , et al. Neuropeptide Y gene therapy decreases chronic spontaneous seizures in a rat model of temporal lobe epilepsy. Brain. 2008;131(Pt 6):1506–1515. 18477594 10.1093/brain/awn079

[19] Melin E Nanobashvili A Avdic U , et al. Disease modification by combinatorial single vector gene therapy: a preclinical translational study in epilepsy. Mol Ther Methods Clin Dev. 2019;15:179–193. 31660420 10.1016/j.omtm.2019.09.004 PMC6807261

[20] Haberman RP Samulski RJ McCown TJ . Attenuation of seizures and neuronal death by adeno-associated virus vector galanin expression and secretion. Nat Med. 2003;9(8):1076–1080. 12858168 10.1038/nm901

[21] Goossens MG Boon P Wadman W , et al. Long-term chemogenetic suppression of seizures in a multifocal rat model of temporal lobe epilepsy. Epilepsia. 2021;62(3):659–670. 33570167 10.1111/epi.16840

[bibr22-15357597231176234] Katzel D Nicholson E Schorge S Walker MC Kullmann DM . Chemical-genetic attenuation of focal neocortical seizures. Nat Commun. 2014;5:3847. 24866701 10.1038/ncomms4847 PMC4050272

[23] Miyakawa N Nagai Y Hori Y , et al. Chemogenetic attenuation of cortical seizures in nonhuman primates. Nat Commun. 2023;14(1):971. 36854724 10.1038/s41467-023-36642-6 PMC9975184

[24] Hristova K Martinez-Gonzalez C Watson TC , et al. Medial septal GABAergic neurons reduce seizure duration upon optogenetic closed-loop stimulation. Brain. 2021;144(5):1576–1589. 33769452 10.1093/brain/awab042 PMC8219369

[bibr25-15357597231176234] Krook-Magnuson E Armstrong C Oijala M Soltesz I . On-demand optogenetic control of spontaneous seizures in temporal lobe epilepsy. Nat Commun. 2013;4:1376. 23340416 10.1038/ncomms2376 PMC3562457

[bibr26-15357597231176234] Krook-Magnuson E Szabo GG Armstrong C Oijala M Soltesz I . Cerebellar directed optogenetic intervention inhibits spontaneous hippocampal seizures in a mouse model of temporal lobe epilepsy. eNeuro. 2014;1(1):ENEURO.0005–14.2014. 25599088 10.1523/ENEURO.0005-14.2014 PMC4293636

[bibr27-15357597231176234] Walker MC Kullmann DM . Optogenetic and chemogenetic therapies for epilepsy. Neuropharmacology. 2020;168:107751. 10.1016/j.neuropharm.2019.107751 31494141

[28] Paz JT Davidson TJ Frechette ES , et al. Closed-loop optogenetic control of thalamus as a tool for interrupting seizures after cortical injury. Nat Neurosci. 2013;16(1):64–70. 23143518 10.1038/nn.3269 PMC3700812

[29] Lieb A Qiu Y Dixon CL , et al. Biochemical autoregulatory gene therapy for focal epilepsy. Nat Med. 2018;24(9):1324–1329. 29988123 10.1038/s41591-018-0103-x PMC6152911

[30] Colasante G Qiu Y Massimino L , et al. In vivo CRISPRa decreases seizures and rescues cognitive deficits in a rodent model of epilepsy. Brain. 2020;143(3):891–905. 32129831 10.1093/brain/awaa045 PMC7089667

[31] Wykes RC Heeroma JH Mantoan L , et al. Optogenetic and potassium channel gene therapy in a rodent model of focal neocortical epilepsy. Sci Transl Med. 2012;4(161):161ra52. 10.1126/scitranslmed.3004190 PMC3605784 23147003

[32] Snowball A Chabrol E Wykes RC , et al. Epilepsy gene therapy using an engineered potassium channel. J Neurosci. 2019;39(16):3159–3169. 30755487 10.1523/JNEUROSCI.1143-18.2019 PMC6468110

[33] Qiu Y O’Neill N Maffei B , et al. On-demand cell-autonomous gene therapy for brain circuit disorders. Science. 2022;378(6619):523–532. 36378958 10.1126/science.abq6656 PMC7613996

[34] Hill SF Ziobro JM Jafar-Nejad P Rigo F Meisler MH . Genetic interaction between Scn8a and potassium channel genes Kcna1 and Kcnq2. Epilepsia. 2022;63(10):e125–e131. 35892317 10.1111/epi.17374 PMC9804156

[35] Wong JC Makinson CD Lamar T , et al. Selective targeting of Scn8a prevents seizure development in a mouse model of mesial temporal lobe epilepsy. Sci Rep. 2018;8(1):126. 29317669 10.1038/s41598-017-17786-0 PMC5760706

[36] Carpenter JC Lignani G . Gene editing and modulation: the holy grail for the genetic epilepsies? Neurotherapeutics. 2021;18(3):1515–1523. 34235638 10.1007/s13311-021-01081-y PMC8608979

[37] Matos L Duarte AJ Ribeiro D Chaves J Amaral O Alves S . Correction of a splicing mutation affecting an Unverricht-Lundborg disease patient by antisense therapy. Genes (Basel). 2018;9(9):455. 30208654 10.3390/genes9090455 PMC6162617

[38] Lim KH Han Z Jeon HY , et al. Antisense oligonucleotide modulation of non-productive alternative splicing upregulates gene expression. Nat Comm. 2020;11(1):3501. 10.1038/s41467-020-17093-9 PMC7347940 32647108

[39] Yeo XY Cunliffe G Ho RC Lee SS Jung S . Potentials of neuropeptides as therapeutic agents for neurological diseases. Biomedicines. 2022;10(2):343. 35203552 10.3390/biomedicines10020343 PMC8961788

[40] Drake CT Terman GW Simmons ML , et al. Dynorphin opioids present in dentate granule cells may function as retrograde inhibitory neurotransmitters. J Neurosci. 1994;14(6):3736–3750. 7911518 10.1523/JNEUROSCI.14-06-03736.1994 PMC6576943

[41] Cattaneo S Verlengia G Marino P Simonato M Bettegazzi B . NPY and gene therapy for epilepsy: how, when,…and Y. Front Mol Neurosci. 2020;13:608001. 33551745 10.3389/fnmol.2020.608001 PMC7862707

[42] Mazarati AM Hohmann JG Bacon A , et al. Modulation of hippocampal excitability and seizures by galanin. J Neurosci. 2000;20(16):6276–6281. 10934278 10.1523/JNEUROSCI.20-16-06276.2000 PMC6772610

[43] Weston M Kaserer T Wu A , et al. Olanzapine: a potent agonist at the hM4D(Gi) DREADD amenable to clinical translation of chemogenetics. Sci Adv. 2019;5(4):eaaw1567. 31001591 10.1126/sciadv.aaw1567 PMC6469940

[44] Berecki G Bryson A Terhag J , et al. SCN1A gain of function in early infantile encephalopathy. Ann Neurol. 2019;85(4):514–525. 30779207 10.1002/ana.25438

[45] Wykes RC Lignani G. Gene therapy and editing: novel potential treatments for neuronal channelopathies. Neuropharmacology. 2018;132:108–117. 28564577 10.1016/j.neuropharm.2017.05.029

[46] Turner TJ Zourray C Schorge S Lignani G . Recent advances in gene therapy for neurodevelopmental disorders with epilepsy. J Neurochem. 2021;157(2):229–262. 32880951 10.1111/jnc.15168 PMC8436749

[47] Wang W Takashima S Segawa Y , et al. The developmental changes of Nav1.1 and Nav1.2 expression in the human hippocampus and temporal lobe. Brain Res. 2011;1389:61–70. 21377452 10.1016/j.brainres.2011.02.083

[48] Valassina N Brusco S Salamone A , et al. Scn1a gene reactivation after symptom onset rescues pathological phenotypes in a mouse model of Dravet syndrome. Nat Commun. 2022;13(1):161. 35013317 10.1038/s41467-021-27837-w PMC8748984

[49] Nabbout R Chemaly N Chipaux M , et al. Encephalopathy in children with Dravet syndrome is not a pure consequence of epilepsy. Orphanet J Rare Dis. 2013;8(1):176. 24225340 10.1186/1750-1172-8-176 PMC4225757

[50] Thijs RD Surges R O’Brien TJ Sander JW . Epilepsy in adults. Lancet. 2019;393(10172):689–701. 30686584 10.1016/S0140-6736(18)32596-0

[51] Martins Custodio H Clayton LM Bellampalli R , et al. Widespread genomic influences on phenotype in Dravet syndrome, a ‘monogenic’ condition. Brain. 2023. 10.1093/brain/awad111 PMC10473570 37006128

